# Total atrial conduction time predicts new-onset atrial fibrillation in patients with transthyretin amyloid cardiomyopathy

**DOI:** 10.1093/europace/euaf164

**Published:** 2025-08-02

**Authors:** Beatrice Dal Passo, Matteo Arzenton, Anna Cantone, Gioele Fabbri, Jacopo Bonini, Gabriele Guidi Colombi, Angelo Melpignano, Federico Marchini, Carmen Izzo, Rita Pavasini, Matteo Bertini, Gianluca Campo, Matteo Serenelli

**Affiliations:** Cardiology Department, Azienda Ospedaliero-Universitaria di Ferrara, Via Aldo Moro 8, 44124 Ferrara (FE), Italy; Cardiology Department, Azienda Ospedaliero-Universitaria di Ferrara, Via Aldo Moro 8, 44124 Ferrara (FE), Italy; Cardiology Department, Azienda Ospedaliero-Universitaria di Ferrara, Via Aldo Moro 8, 44124 Ferrara (FE), Italy; Cardiology Department, Azienda Ospedaliero-Universitaria di Ferrara, Via Aldo Moro 8, 44124 Ferrara (FE), Italy; Cardiology Department, Azienda Ospedaliero-Universitaria di Ferrara, Via Aldo Moro 8, 44124 Ferrara (FE), Italy; Cardiology Department, Azienda Ospedaliero-Universitaria di Ferrara, Via Aldo Moro 8, 44124 Ferrara (FE), Italy; Cardiology Department, Azienda Ospedaliero-Universitaria di Ferrara, Via Aldo Moro 8, 44124 Ferrara (FE), Italy; Cardiology Department, Azienda Ospedaliero-Universitaria di Ferrara, Via Aldo Moro 8, 44124 Ferrara (FE), Italy; Cardiology Department, Azienda Ospedaliero-Universitaria di Ferrara, Via Aldo Moro 8, 44124 Ferrara (FE), Italy; Cardiology Department, Azienda Ospedaliero-Universitaria di Ferrara, Via Aldo Moro 8, 44124 Ferrara (FE), Italy; Cardiology Department, Azienda Ospedaliero-Universitaria di Ferrara, Via Aldo Moro 8, 44124 Ferrara (FE), Italy; Cardiology Department, Azienda Ospedaliero-Universitaria di Ferrara, Via Aldo Moro 8, 44124 Ferrara (FE), Italy; Cardiology Department, Azienda Ospedaliero-Universitaria di Ferrara, Via Aldo Moro 8, 44124 Ferrara (FE), Italy

**Keywords:** Transthyretin amyloid cardiomyopathy, Atrial fibrillation, PA-TDI

Transthyretin amyloid cardiomyopathy (ATTR-CM) is an infiltrative cardiomyopathy resulting from age-related failure of homoeostatic mechanisms in wild-type ATTR (wtATTR-CM) or destabilizing mutations in variant TTR (vATTR-CM).^1^ Atrial fibrillation (AF) is very prevalent in ATTR-CM, affecting up to 70% of patients.^2^ Due to significant diastolic dysfunction, the loss of atrial contribution to ventricular filling in AF is harmful and associated with clinical deterioration.^2^ Management of AF in ATTR-CM is challenging because rate-control medications are poorly tolerated and data on rhythm control strategies is limited.^2^ Kanazawa e*t al*. recently reported 44% of recurrence-free rate of AF, atrial flutter, and atrial tachycardia after catheter ablation (CA) at 5 years follow-up in a cohort of 54 patients with ATTR-CM. However, CA demonstrated to significantly reduce heart failure (HF) hospitalization and consequently cardiovascular mortality tended to be lower.^3^

Total atrial conduction time estimated by tissue-Doppler imaging (PA-TDI) is an echocardiography-derived parameter consisting of the time needed for the atrial depolarization to occur (ECG P-wave) and resulting in active atrial contraction (TDI A′-wave). It reflects LA electrical and structural changes being inversely related to LA indexed volume (LAVi) and LA reservoir strain (LASr).^4,5^ It also predicts new-onset AF (NOAF) in various populations.^4,5^ Still, it remains unexplored in ATTR-CM.

The main object of this study is to define whether PA-TDI predicts NOAF in ATTR-CM. All patients attending our amyloidosis clinic gave written informed consent and were enrolled in the AMI-ER registry (107/2024/Oss/AOUFe). We prospectively collected echocardiographic data of subjects with a confirmed diagnosis of ATTR-CM from October 2021 to June 2024. We enrolled patients with no previous diagnosis of AF and a clinical follow-up of at least six months. The primary endpoint was the occurrence of NOAF. Diagnosis was defined by clinical assessment and confirmed by a 12-lead ECG. Baseline echocardiogram comprehended dimensions of cardiac chambers, filling pressure of the left ventricle (LV), LV global longitudinal strain (avGLS), LA strain analysis, and PA-TDI, which was measured as the time interval from the beginning of the P-wave on the ECG to the peak A′-wave on the tissue-Doppler imaging (TDI) tracing of the lateral LA wall on echocardiography.^4,5^ The examinations were analysed blindly by a cardiologist with expertise in cardiac imaging and cardiomyopathy. A total of 112 patients were screened. Seventy-three had a history of AF, and seven had echocardiographic images unsuitable for PA-TDI calculation. Thirty-two subjects were enrolled. Twenty-four subjects had wtATTR-CM, and eight had vATTR-CM; subjects were predominantly male (80%), and the median age was 78.7 (IQR 72.6–82.3) years. After a median follow-up of 32 months, six (19%) subjects developed NOAF. Baseline patient characteristics are shown in *Table 1*. Those who developed NOAF shared similar clinical features to patients who did not (*Table 1*). There was no difference in LV grade of hypertrophy and function. Subjects who developed AF had significantly larger LAVi [(50.5 mL/m^2^ vs. 36.0 mL/m^2^, *P*-value 0.014)]. Still, we did not observe differences in terms of LASr [(14.0% vs. 7.7%, *P*-value 0.64)], left atrial contraction strain (LASct) [(7.0% vs. 5.0%, *P*-value 0.74)], and left atrial conduit strain (LAScd) [(7.1% vs. 4.0%, *P*-value 0.34)]. PA-TDI was longer in patients who developed AF [(196.83 ms vs. 150.0 ms, *P* < 0.001)] (*Figure 1*). At Kaplan–Meyer analysis, a PA-TDI > 170 ms was associated with an HR of 4.48 of NOAF (log-rank *P* < 0.001). The cut-off was determined by the Liu method. At Cox regression analysis, LAVi (HR 1.01 for 1 mL/m^2^ increase; 95% CI 1.02–1.12), LV indexed mass (HR 1.02 for 1 gr/m^2^ increase; 95% CI 1.00–1.03), and PA-TDI (HR 1.05 for 1 ms increase; 95% CI 1.01–1.09) were significantly associated with NOAF. The PA-TDI remained an independent predictor of NOAF in three different multivariable model testing LAVi (HR 1.04; 95% CI 1.01–1.09), LV indexed mass (HR 1.05; 95% CI 1.01–1.12) and LASr (HR 1.05; 95% CI 1.01–1.10) as covariates.

**Figure 1:**
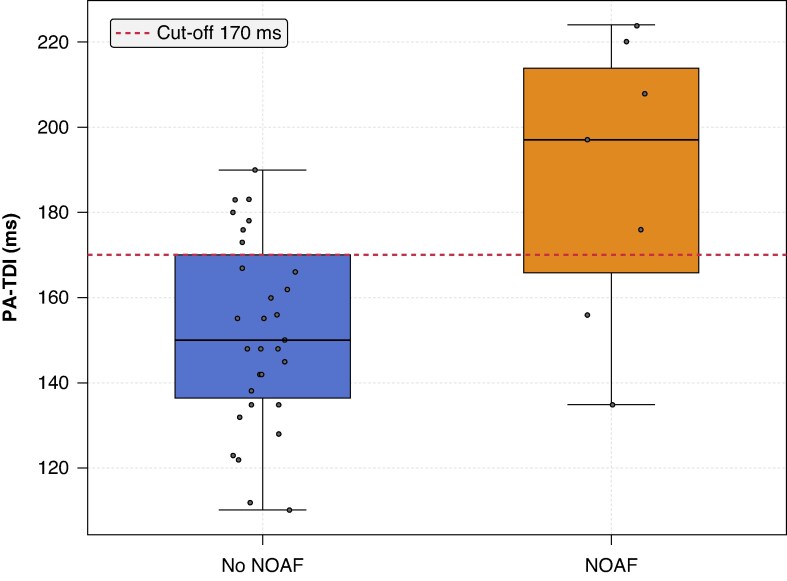
Box plot showing the median and interquartile range of the distribution of PA-TDI values according to the development of now-onset atrial fibrillation. NOAF, new-onset atrial fibrillation. Black dots represent the value of PA-TDI for each observation.

**Table 1:** Epidemiological and echocardiographic characteristics of the population

	Total population (*n* = 32)	No new-onset atrial fibrillation (*n* = 26)	New-onset atrial fibrillation (*n* = 6)	*P*-value
Clinical assessment				
vATTR-CM (*n*, %)	8 (25)	8 (31)	0 (0)	<0.001
Age (years, IQR)	78.7 (72.6–82.3)	78.2 (69.2–80.8)	83.4 (74.6–86.3)	0.15
Male (*n*, %)	24 (80)	19 (79)	5 (83)	1.00
BMI (kg/m^2^, IQR)	26.5 (23.5–29.0)	26.5 (23.0–29.0)	27.0 (25.0–29.0)	0.63
Hypertension (*n*, %)	24 (75)	20 (77)	4 (67)	0.62
Diabetes mellitus (*n*, %)	6 (19)	5 (19)	1 (17)	1.00
Echocardiography				
IVS (mm, ±DS)	16.2 (±2.1)	16.0 (±2.0)	16.8 (±2.2)	0.38
LV indexed mass (g/m^2^, ±DS)	153.5 (±31.6)	149.3 (±30.9)	175.2 (±28.8)	0.094
LAV indexed (mL/m^2^, IQR)	38.8 (33.5–50.0)	36.0 (30.0–45.0)	50.5 (43.0–64.0)	0.014
RAV indexed (mL/m^2^, IQR)	35.0 (24.5–47.0)	30.0 (23.0–40.0)	50.0 (41.0–53.0)	0.067
E/e′ (ratio, IQR)	15.9 (11.9–21.2)	15.9 (11.8–20.9)	17.5 (12.5–26.3)	0.63
avGLS (%, IQR)	−12.9 (−14.1–10.4)	−13.0 (−14.1–11.1)	−10.7 (−13.0–9.6)	0.24
LAS reservoir (%, IQR)	13.0 (9.0–19.4)	14.0 (10.2–18.8)	7.7 (6.7–23.0)	0.64
LAS contraction (%, IQR)	7.0 (5.0–10.0)	7.0 (5.1–9.5)	5.0 (3.0–11.0)	0.74
LAS conduit (%, IQR)	7.0 (4.0–9.8)	7.1 (5.4–9.8)	4.0 (2.7–8.0)	0.34
PA-TDI interval (ms, ±DS)	159.7 (29.5)	150.0 (21.8)	196.8 (26.6)	<0.001

vATTR-CM, variant amyloid cardiomyopathy; BMI, body mass index; IVS, interventricular septum end-diastolic thickness; LV, left ventricular; LAV, left atrial volume; RAV, right atrial volume; avGLS, average global longitudinal strain; LAS, left atrial strain; PA-TDI, total atrial conduction time.

This is the first study quantifying PA-TDI in a cohort of patients with ATTR-CM. LAVi and all three components of LAS were markedly pathological in our cohort. In addition, even if a ‘normal’ value for PA-TDI hasn’t been established yet, our cohort’s mean PA-TDI value of 159.7 ± 29.5 ms was significantly longer than that found in healthy subjects and in other cardiopathies,^4,5^ including hypertrophic cardiomyopathy (115.2 ± 29.8 ms) and cardiac sarcoidosis (67.9 ± 16.1 ms).^6,7^

These data support the critical atrial involvement and significant electromechanical dysfunction in ATTR-CM. Previous echocardiographic and anatomopathological data showed atrial dysfunction due to significant amyloid infiltration in the atria that causes loss of normal architecture and up-regulation of the collagen, rather than secondary atrial dysfunction due to increased LV filling pressure.^8^ In particular, Bandera *et al*. firstly showed, in a broad cohort of ATTR-CM patients, a high prevalence of patients with increased atrial stiffness (expressed as the ratio between E/e′ and LASr), reduced LASr, LAScd, and LASct that leads to define a clinical phenotype characterized by atrial electromechanical dissociation despite the evidence of sinus rhythm on the ECG. Furthermore, these patients were characterized by poor prognosis.^8^

This is consistent with the recent Clinical Consensus Statement from the European Heart Rhythm Association (EHRA), which identifies amyloidogenesis as one of the pathophysiological mechanisms underlying atrial cardiomyopathy—whether in the context of transthyretin (TTR)-related or light-chain (AL)-related amyloid cardiomyopathy, or as an isolated condition, such as isolated atrial amyloidosis (IAA).^9^

In our analysis, the PA-TDI emerged as an independent predictor of NOAF, carrying critical prognostic implications. Despite already being reported in other settings, we did not find LAS to be predictive of outcome.^10^ This could be explained by the ‘floor effect’, being all LAS components hugely depressed in our population.

This study has limitations: the retrospective and monocentric design and the small number of subjects. Finally, it was impossible to analyse the PA-TDI in some patients because of suboptimal ECG traces.

In conclusion, our data show that PA-TDI is an addictive prognostic marker for NOAF over other echocardiographic measurements, such as LAVi or LAS.

## Data Availability

The data underlying this article will be shared on reasonable request to the corresponding author.
